# Deletion at chromosome arm 9p in relation to *BRAF*/*NRAS* mutations and prognostic significance for primary melanoma

**DOI:** 10.1002/gcc.20753

**Published:** 2010-05

**Authors:** Caroline Conway, Samantha Beswick, Faye Elliott, Yu-Mei Chang, Juliette Randerson-Moor, Mark Harland, Paul Affleck, Jerry Marsden, D Scott Sanders, Andy Boon, Margaret A Knowles, D Timothy Bishop, Julia A Newton-Bishop

**Affiliations:** 1 Section of Epidemiology and Biostatistics, Leeds Institute of Molecular Medicine, University of Leeds, St. James's University HospitalLeeds, UK; 2 Department of Dermatology, University Hospital Birmingham NHS Foundation TrustUK; 3 Department of Histopathology, Coventry and Warwickshire PathologyLakin Road, Warwick, UK; 4 Department of Histopathology, Leeds Teaching Hospitals NHS Trust, St. James's University HospitalLeeds, UK; 5 Section of Experimental Oncology, Leeds Institute of Molecular Medicine, University of Leeds, St. James's University HospitalLeeds, UK

## Abstract

We report an investigation of gene dosage at 9p21.3 and mutations in *BRAF* and *NRAS*, as predictors of relapse and histological markers of poor melanoma prognosis. Formalin-fixed primary melanomas from 74 relapsed and 42 nonrelapsed patients were sequenced for common *BRAF* and *NRAS* mutations (*N* = 71 results) and gene dosage at 9p21.3 including the genes *CDKN2A* (which encodes CDKN2A and P14ARF), *CDKN2B* (CDKN2B), and *MTAP* was measured using multiplexed ligation-dependant probe amplification (MLPA), (*N* = 75 results). *BRAF*/*NRAS* mutations were detected in 77% of relapsers and 58% of nonrelapsers (Fisher's exact *P* = 0.17), and did not predict ulceration or mitotic rate. There was no relationship between *BRAF*/*NRAS* mutations and gene dosage at 9p21.3. Reduced gene dosage at *MTAP* showed a borderline association with *BRAF* mutation (*P* = 0.04) and reduced gene dosage at the interferon gene cluster was borderline associated with wild type *NRAS* (*P* = 0.05). Reduced gene dosage in the *CDKN2A* regions coding for CDKN2A was associated with an increased risk of relapse (*P* = 0.03). Reduced gene dosage across 9p21.3 was associated with increased tumor thickness, mitotic rate, and ulceration (*P* = 0.02, 0.02, and 0.002, respectively), specifically in coding regions impacting on CDKN2B and P14ARF and CDKN2A. Loss at *MTAP* (*P* = 0.05) and the interferon gene cluster (*P* = 0.03) on 9p21 was also associated with tumor ulceration. There was no association between reduced gene dosage at 9p21.3 and subtype or site of tumor. This study presents supportive evidence that CDKN2B, P14ARF, and CDKN2A may all play a tumor suppressor role in melanoma progression. © 2010 Wiley-Liss, Inc.

## INTRODUCTION

The AJCC staging system for melanoma utilizes the Breslow thickness of primary melanoma, the presence of ulceration, and staging by sentinel node biopsy to give the best estimate of prognosis (Balch et al.,^2001^). Use of the staging system is associated with a range of estimates of outcome (Balch et al.,^2001^; Gimotty et al.,^2005^), so that a significant proportion of the variance in survival remains unexplained. Therefore, it is important to seek molecular markers of metastatic potential for use in clinical practice. In recent years, progress has taken place in understanding the somatic events that occur in melanoma primary tumors. However, there is comparatively little known of the correlation between these events and outcome, and it is therefore important to increase our understanding of the carcinogenetic process so that logical approaches to treatment can be developed.

A number of signal transduction and cell cycle regulatory pathways have been implicated in the etiology and progression of melanoma, including the retinoblastoma (RB1) and p44/42 mitogen-activated protein kinase (MAPK) pathways. A key region is 9p21.3, which contains the *CDKN2A* and *CDKN2B* genes. These genes encode three separate tumor suppressor proteins. *CDKN2A* encodes both CDKN2A and, using a separate first exon and alternate reading frame, P14ARF. Although both transcripts use exons 2 and 3 of *CDKN2A*, the CDKN2A and P14ARF proteins share no homology at the amino acid level and have distinct tumor suppressor functions in the RB1 and TP53 pathways; *CDKN2B* encodes CDKN2B, which has its own open reading frame (Sharpless and DePinho,^1999^; Weber et al.,^1999^). The CDKN2A protein controls passage through the G1 checkpoint of the cell cycle by inhibiting the phosphorylation of the RB1 protein (Roussel,^1999^) and of the three tumor suppressors at this locus is the one longest recognized to have a significant role in melanoma. It is known to play a key role in normal melanocyte senescence (Ha et al.,^2008^). The P14ARF protein acts on the TP53 cell cycle control pathway by interaction with the human double minute (HDM2) protein to stabilize TP53 and allow cell cycle arrest at the G1/G2 phase (Weber et al.,^1999^). *CDKN2A* was identified first as a tumor suppressor gene commonly deleted/mutated in tumor cell lines (Kamb et al.,1994a) and subsequently its role as a high-risk susceptibility gene in melanoma families was elucidated (Kamb et al.,1994b). Germline mutations have been identified in ∼20% of tested melanoma families (Goldstein and Tucker,^2001^; Bishop et al.,^2002^). Some mutations impact on CDKN2A protein alone, some on P14ARF, and some on both proteins.

Most melanoma cell lines show deletion/mutation of *CDKN2A* (Flores et al.,^1996^; Walker et al.,^1998^). The majority of primary tumors have allelic loss at microsatellite markers mapping to the *CDKN2A* locus, indicating that deletions are the principal genetic event in vivo (Flores et al.,^1996^; Rodolfo et al.,^2004^). More recent reports showed biallelic deletion in ∼45% of melanoma metastases, supporting the role of this locus in melanoma progression (Grafstrom et al.,^2005^) and we have shown that epigenetic silencing of P14ARF is also common in metastatic disease (Freedberg et al.,^2008^). There are few data from primary tumors on the role of deletion at the locus on outcome (Koynova et al.,^2007^) and none on the effect of deletion across the larger region, which we have addressed using Multiplex ligation-dependent probe amplification (MLPA) (Nygren et al.,^2005^) rather than by studying the small intragenic regions previously reported.

More recently there has been some suggestion that CDKN2B may also have tumor suppressor functions in melanoma. Krimpenfort et al. (2007) showed that mice null for CDKN2B, CDKN2A, and P14ARF were more tumor prone than CDKN2A/P14ARF null mice and developed a predominance of skin tumors (Krimpenfort et al.,^2007^). The authors suggested that CDKN2B might act as critical “back up” tumor suppressor in cells null for *CDKN2A*.

Activating mutations of the *NRAS* and *BRAF* genes occur in ∼20 and 50% of malignant melanomas respectively, and are almost always mutually exclusive (Omholt et al.,^2003^; Garnett and Marais,^2004^). *BRAF* and *NRAS* mutations have also been found in benign nevi (Poynter et al.,^2006^) and are therefore thought to be involved early in melanoma carcinogenesis. In cultured human melanocytes, mutant BRAF protein has been shown to induce cell senescence by increasing the expression of CDKN2A (Michaloglou et al.,^2005^). It is postulated, therefore, that to become an invasive melanoma, arrest of the cell cycle caused by normal CDKN2A must subsequently be overcome by mutation or deletion of *CDKN2A* or by alterations to other cell cycle regulators. Moreover, a recent in vitro study showed that simultaneous knockdown of *BRAF* and expression of CDKN2A in melanoma cells led to potent growth inhibition and apoptosis, whereas knockdown of *BRAF* or expression of CDKN2A alone did not (Zhao et al.,^2008^). Studies of *BRAF* mutated nevi using the senescence marker SA-β-gal, however, revealed a marked mosaic induction of CDKN2A, which the authors suggested was indicative of a role for multiple tumor suppressors in the prevention of *BRAF* oncogenesis (Michaloglou et al.,^2005^).

In this study, we have investigated the gene dosage of multiple tumor suppressors at 9p21 in formalin-fixed, paraffin-embedded (FFPE) primary melanoma tumors. Furthermore, we have investigated the relationship between reduced gene dosage at the *CDKN2A* locus and *BRAF/NRAS* mutations using tumors from patients who have relapsed and from patients with similar tumors who have not relapsed, to determine the prognostic value of these events. The presence of ulceration is an important prognostic factor for melanoma even in stage III or metastatic disease (Balch et al.,^2001^) and, therefore, we also assessed the associations between *CDKN2A* deletion and *BRAF/NRAS* mutation and ulceration. In other series, mitotic rate has important prognostic significance (Elder and Murphy,^2008^), and therefore, we also examined genetic markers in relation to mitotic rate. CDKN2A is a cyclin D kinase (CDK) inhibitor and therefore loss of CDKN2A would likely be related to increased mitotic rate.

The methylthioadenosine phosphorylase (*MTAP*) gene is also located at 9p. It has been suggested that loss of expression of the gene has prognostic implications for melanoma (Behrmann et al.,^2003^) and codeletion of *MTAP* with *CDKN2A* has been investigated in a number of cancers (Chen et al.,^1996^). There is evidence that loss of *MTAP* results in an inhibition of STAT signalling pathways regulated by interferon, so it is of interest that response of melanoma patients to interferon used as an adjuvant therapy for this cancer has been reported to be related to *MTAP* status (Wild et al.,^2007^). We were able in this study to look at deletion of *MTAP* in primary melanomas.

## MATERIALS AND METHODS

### Patients

Ethical approval for this study was obtained from the Multi-Regional Ethical Committee (MREC) and from the patient information advisory group (PIAG) and all living patients gave informed written consent to the use of their stored tissues for research. Melanoma cases had all been diagnosed at least 3 years previously. Recruitment was irrespective of family history. Cases were then eligible as “relapsers” if relapse occurred after 3 years or as “nonrelapsers” if they were free of relapse. Participants had a tumor thickness greater than 0.75 mm and were recruited between May 2000 and January 2005. Full details of the study were reported previously (Beswick et al.,^2008^). There were 424 patients eligible for the study, of which 66% (278) participated. The median Breslow thickness was 1.6 mm (range 0.8-20). From the 278 participating patients, 116 (74 relapsers and 42 nonrelapsers) had FFPE primary tumor samples that were available for further sampling and DNA extraction. There was no selection of these blocks, other than that we used blocks which could be traced.

### Histology

Sections from the primary tumors were examined (blind to relapse status) by one pathologist according to protocol (AB). The following were recorded: Breslow thickness, site of the primary tumor, histological sub-type, presence of ulceration, and mitotic rate in three categories (0, 1 to 6 and more than 6 per mm^2^).

### DNA Extraction

DNA was extracted from 116 FFPE primary melanoma tumors, sampled horizontally at the advancing edge of the tumor, in the vertical growth phase, using a 0.8 mm × 2 mm core biopsy needle and haematoxylin and eosin stained slides as a guide. The intent was to choose tissue representative of the deepest part of the tumor but which was sufficiently surrounded by tumor that the sample contained minimal normal stroma and inflammatory cell infiltrate. Horizontal sections of cores were taken during development of the methodology to ensure the technique allowed minimal sampling of normal tissue as described previously (Conway et al.,^2009^). DNA extraction from cores was carried out using the QIAamp DNA Mini kit (Qiagen, Sussex, UK) (Conway et al.,^2009^).

### Copy Number Analysis of Chromosome 9p21

**Figure 1:**
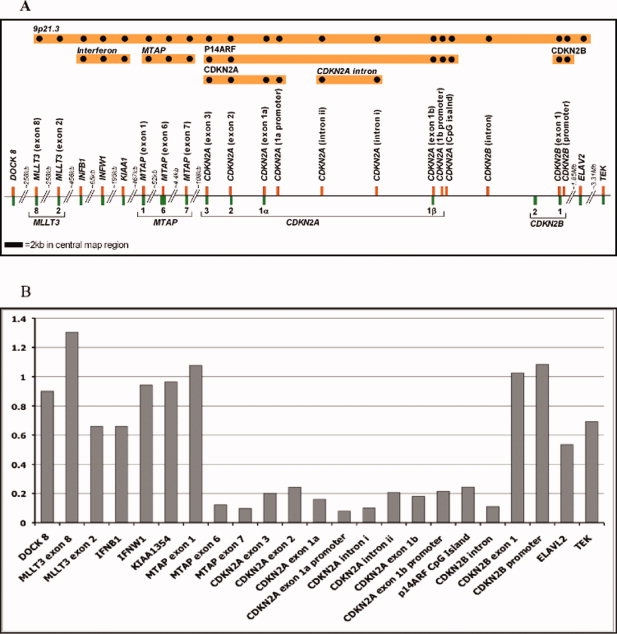
MLPA for analysis of gene dosage at chromosome 9p21. A, a diagrammatical representation of the 9p21 region covered by the P024A MLPA kit. Locations of probed genes are shown in green and MLPA probes in orange with probe names outlined in bold. Probe groupings used in data analysis are represented by orange bars with black dots at top of the picture. B, gene dosage ratios for a representative primary melanoma sample showing an almost total loss of gene dosage (representing ≥80% loss) from MTAP exon 6 to *CDKN2B* intron.

Gene dosage ratios for 12 *CDKN2A/CDKN2B* locus sites and 11 other 9p gene sites were determined using the 9p21 MLPA kit (P024 MRC-Holland, Amsterdam, the Netherlands) (Schouten et al.,^2002^). The genes included in this screen were *TEK, ELAV2, CDKN2B, CDKN2A, MTAP, KIAA1354, INFW1, INFB1, MLLT3*, and *DOCK8* (Fig. 1A). The kit was used in accordance with instructions for all experiments but in 1/4 volumes of those recommended by the supplier, based on previous optimization in our laboratory: 30–100 ng of extracted tumor DNA was denatured and target gene probes were hybridized to the target DNA prior to probe ligation in the presence of ligase-65. The ligation products were subject to polymerase chain reaction (PCR) amplification performed on a GeneAmp PCR System 9700 Thermal Cycler (Applied Biosystems, Warrington, UK) with a hot-start PCR program. MLPA fragments were visualized on an ABI 3130XL Automated DNA Sequencer with a 36 cm capillary array, ABI POP-7 polymer, and GeneScan-ROX 500 size standards (Applied Biosystems). Peak detection analysis has been automated using ABI PRISM Genescan® Analysis software version 3.1 (Applied Biosystems) and GeneMarker software (Softgenetics, State College, Pennsylvania, USA). In each set of experiments, one negative control (no DNA) sample and four normal control samples (human genomic DNA with normal gene dosage at 9p from four different individuals) were included.

### MLPA Data Analysis

Gene dosage analysis was automated using the MLPA analysis program included with GeneMarker software version 1.6 (Softgenetics, Pennsylvania, USA) according to the manufacturer's instructions. Data were normalized using the “population normalization” mode (as recommended by the MLPA kit manufacturers for analysis of tumor DNA). Peak heights were normalized according to the median height of all test and control peak heights of similar fragment size. Normalized peak heights were then compared to a synthetic control sample (average of peak heights from four human genomic DNA samples with normal gene dosage at 9p) with default analysis parameters to determine gene dosage ratios where the median point within the data set is considered to be 1, and a gene dosage ratio for each probe region was calculated with reference to the synthetic control (Fig. 1B). In previous publications using MLPA on FFPE-derived DNA, gene dosage cut-offs of 0.7 for loss and 1.3 for gain have been used according to manufacturer's instructions (van Dijk et al.,^2005^; Takata,^2008^; Buffart et al.,^2009^) (and author correspondence with MRC-Holland). In our analysis, gene dosage was treated as a continuous variable to account for the effect of any possible contaminating normal DNA.

### Mutation Analyses

Gene fragments of hotspot mutation regions in *BRAF* (exon 15) and *NRAS* (exon 2) were amplified by PCR in separate reactions to screen for common mutations found in melanomas. Standard PCR reactions were carried out using Amplitaq Gold DNA polymerase in 1× PCR buffer (Applied Biosystems) according to manufacturer's instructions. Primers: *BRAF* exon 15 Forward: 5′-TCA TAATGCTTGCTCTGATAGGA and Reverse: 5′-GGCCAAAAATTTAATCAGTGGA (annealing temperature 59°C); and *NRAS* exon 2 Forward: 5′-GGTGAAACCTGTTTGTTGGA and Reverse: 5′-TTCAGAACACAAAGATCATC (55°C).

*BRAF* and *NRAS* PCR products were sequenced in both directions using an ABI3100 Automated DNA Sequencer with a 36 cm capillary array, ABI POP-7 polymer and ABI Prism BigDye Terminator Cycle Sequencing Kit version 1.1 (Applied Biosystems) according to the manufacturer's instructions. Sequence analysis was carried out using CodonCode Aligner sequencing software (CodonCode Corporation, Dedham, MA, USA) and mutation detection was based on *BRAF* and *NRAS* cDNA sequences (Genbank accession nos. NM_004333 and NM_00254, respectively).

### CDKN2A Immunohistochemistry

A representative set of 17 tumors from the MLPA data set were selected for immunohistochemistry using antibodies to CDKN2A to evidence the validity of the MLPA results. The samples chosen consisted of four samples with gene dosage ratios of ≤0.2 (80% gene dosage loss) at the *CDKN2A* promoter, or coding regions for CDKN2A; seven samples with gene dosage ratios ∼0.5 (50% gene dosage loss) at the *CDKN2A* promoter, coding regions for CDKN2A or intronic regions upstream of the promoter; and six samples with gene dosage ratios between 0.7 and 1.3 (normal gene dosage). Positive and negative controls for kit and antibody performance were included. The positive control was a paraffin-embedded section of bladder carcinoma with high CDKN2A expression determined by Western blot analysis. The negative control was a section from the same bladder tumor without the addition of primary antibody.

Sections (5 μm) were cut and fixed on Superfrost plus glass slides before dewaxing and rehydration. Expression of CDKN2A was examined using a CDKN2A monoclonal antibody (1:1500 for 1 hr; Ab-7; Labvision, Freemont, CA, USA) and the catalyzed signal amplification system (CSA system; DakoCytomation, Cambridgeshire, UK) according to the manufacturer's instructions. Endogenous biotin or biotin-binding proteins were blocked using the Avidin Biotin blocking kit (Vector Laboratories, Peterborough, UK) according to manufacturer's instructions and endogenous peroxidase activity was blocked using 3% hydrogen peroxide in water for 5 min (CSA system). Sections were counterstained with hematoxylin, dehydrated and mounted in Depex mounting medium (VWR International, Leicestershire, UK). Expression of CDKN2A was examined by light microscopy and scored in tumors as absent (0), expressed (1) or highly expressed (2).

### Statistical Methods

Gene dosage was treated as a continuous variable in the analysis (Fig. 1). To investigate the effect of dosage in relation to each of the transcripts at 9p21, five separate probe groups were created. Probe groups contained all probes located within the coding exons and promoter for each transcript. The probe groups were: (1) *CDKN2B* (probes: *CDKN2B* promoter and exon 1), (2) regions coding for P14ARF (probes: *CDKN2A* CpG island, *CDKN2A* 1β promoter and exons 1β, 2, and 3), (3) CDKN2A (probes: *CDKN2A* 1α promoter and exons 1α, 2, and 3), (4) *MTAP* (probes: *MTAP* exons 1, 6, and 7), and (5) the interferon gene cluster (probes: *KIAA1354* [between *IFNA5* and *IFNA6*], *INFW1*, and *INFB1*). A “*CDKN2A*” group containing all *CDKN2A* probes was created to investigate the effect of overall loss at the *CDKN2A* locus (probes: *CDKN2A* CpG island, *CDKN2A* 1β promoter, exon 1β, *CDKN2A* introns 1 and 2, *CDKN2A* 1α promoter plus exons 1α, 2 and 3). To investigate the effect of loss across the whole 9p21.3 region, an overall 9p21.3 group defined by all of the probes in the region was also considered. Median gene dosage ratio was used to represent the overall gene dosage ratio of the region where there was more than one probe within the region.

A rolling average heatmap was used to represent graphically the overall gene loss. The rolling average of the gene dosage was calculated as the average gene dosage ratio of the probe itself and adjacent probes. The Wilcoxon two-sample rank test using normal approximation was performed to assess the difference between gene dosage ratio by relapse status (relapse versus no relapse), ulceration status (yes versus no), tumor site (head/neck/foot/hand versus others) and *BRAF* and *NRAS* mutation status. The Kruskal Wallis test was applied to assess the difference between tumor histological sub-type (superficial spreading versus nodular) and mitotic rate per mm^2^ (grouped as 0, 1–6, >6). Spearman correlations (r) were used to assess association between gene dosage ratio and Breslow thickness. Fisher's exact test was used to assess the association between *BRAF*/*NRAS* mutation and relapse status, mitotic rate and ulceration. These analyses were carried out using the SAS/STAT statistical software version 9.1 for PC (Copyright, SAS Institute Inc. Cary, NC, USA). The rolling average heatmap was illustrated using heatmap.2<gplots> function in R version 2.9.0 (Vienna, Austria).

## RESULTS

### Samples

In total, 116 primary tumor blocks from 116 patients (74 relapsers and 42 non-relapsers) were sampled for DNA extraction and molecular analysis. The median DNA concentration was 28 ng/μl (range 4–493 ng/μl) with elution volumes of 25 μl resulting in a median total yield of 0.7 μg/tumor. The median tumor block age was 11 years (range 5-36 years). From the 116 tumor blocks sampled, 50 produced DNA of sufficient quality for both MLPA and *BRAF*/*NRAS* analysis, 25 produced results for MLPA only, 21 produced results for *BRAF*/*NRAS* only and 20 did not produce any results. Therefore, the success rate for MLPA was 65% (75/116) and that for *BRAF/NRAS* was 61% (71/116). There was no significant difference in the distribution of block age between samples with a MLPA result (median 10 years, range 5–36) and those that failed (median 12 years, range 5–24), *P* = 0.3 (Wilcoxon rank test). The strongest predictor of assay success was DNA quantity. Failure was much more likely to occur in assays with <100 ng DNA input (44% fail rate at <100 ng DNA compared to 17% fail rate at >100 ng DNA), according to the DNA quantity control peaks within each MLPA assay.

### Gene Dosage at 9p21

**Figure 2:**
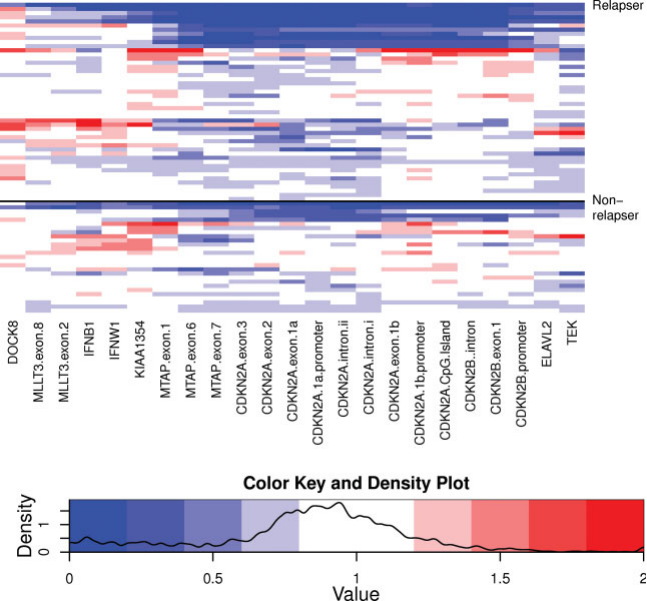
Heatmap of gene dosage at chromosome region 9p21 by relapse status. Gene dosage ratios are shown for tumors from 45 relapsed patients (above) compared with tumors from 27 patients who had not relapsed. Blue coloration indicates reduced gene dosage, red coloration indicates increased gene dosage.

Gene dosage at 9p21 was successfully measured in 75 vertical growth phase primary melanomas (48 relapsers and 27 non-relapsers) using MLPA. Gene dosage ratios for 12 *CDKN2A/CDKN2B* locus sites and 11 other 9p gene sites were determined. Reduced gene dosage was more frequent in relapsers (Fig. 2). It can be seen that although loss of gene dosage occurred across 9p (especially in relapsers) the most common region of loss was between *CDKN2A* exon 1α (coding for CDKN2A) and *MTAP*. The median gene dosage at eight key regions across 9p, and their association with relapse or histological indicators of poor prognosis are presented in Table 1. Relapse was associated with loss at 9p21.3 overall (*P* = 0.04) and loss in regions coding for CDKN2A (one sided *P* = 0.03, two sided *P* = 0.05), and (borderline) associated with loss anywhere in the *CDKN2A* region (from *CDKN2A* CpG island probe to *CDKN2A* exon 3 probe) (one-sided *P* = 0.05, two-sided *P* = 0.1) but not elsewhere across 9p.

**TABLE 1:** Median Gene Dosage by Probe Categories and Indicators of Relapse

	Y = yes N = no	Total numbers	*CDKN2B*	*CDKN2B* (Intron)	*CDKN2A* p14^ARF^	*CDKN2A* p16 Intron	*CDKN2A* p16	All probes within *CDKN2A*	*MTAP*	Interferon	9p21.3
Relapse	Y	48	0.92	0.74	0.94	0.67	0.76	0.82	0.71	0.86	0.85
	N	27	0.88	0.78	0.98	0.77	0.84	0.91	0.77	0.90	0.90
*P*-value (one-sided)			0.47	0.47	0.10	**0.06**	**0.03**	**0.05**	0.26	0.13	**0.04**
Ulceration	Y	29	0.77	0.67	0.82	0.67	0.70	0.77	0.67	0.84	0.81
	N	46	0.96	0.78	0.98	0.73	0.82	0.91	0.77	0.90	0.92
*P*-value (one-sided)			**0.006**	**0.04**	**0.002**	0.11	**0.02**	**0.02**	**0.05**	**0.03**	**0.002**
Breslow thickness	<2 mm	18	1.04	0.86	0.97	0.75	0.84	0.91	0.8	0.89	0.92
	>2<4 mm	30	0.90	0.74	0.97	0.70	0.84	0.86	0.77	0.90	0.88
	>4mm	27	0.79	0.67	0.84	0.70	0.76	0.77	0.67	0.83	0.81
r_s_ (*P*-value)*			**−0.31 (0.007)**	−0.14 (0.22)	**−0.29 (0.01)**	−0.06 (0.58)	**−0.28 (0.01)**	**−0.24 (0.04)**	−0.19 (0.11)	−0.11 (0.34)	**−0.27 (0.02)**
Mitotic rate	0/ mm^2^	3	1.11	0.92	1.01	0.92	0.99	0.99	0.82	0.59	0.96
	1–6/ mm^2^	52	0.93	0.77	0.97	0.72	0.81	0.91	0.75	0.89	0.89
>6/ mm^2^		20	0.59	0.64	0.67	0.57	0.55	0.64	0.69	0.84	0.80
*P*-value**			**0.02**	0.28	**0.0006**	0.28	**0.004**	**0.005**	0.37	0.12	**0.02**
Tumour site	Head/neck/ hand/foot	15	1.06	0.74	1.00	0.85	0.80	0.84	0.69	0.88	0.88
	Other	60	0.90	0.76	0.93	0.69	0.78	0.85	0.76	0.88	0.87
*P*-value (one-sided)			0.12	0.21	0.26	0.30	0.49	0.42	0.24	0.08	0.47
Melanoma type (Wilcoxon two-sample tests were made between nodular and superficial spreading melanomas)	Acral Lentiginous	2	0.44	0.37	0.76	0.53	0.61	0.62	0.44	0.67	0.58
	Desmoplastic	1	1.10	0.73	0.82	1.02	0.80	0.83	0.69	0.97	0.97
	Indeterminate	3	1.06	0.11	0.22	0.10	0.18	0.20	0.12	0.97	0.23
	Nodular	28	0.88	0.78	0.93	0.72	0.77	0.81	0.67	0.88	0.83
	Superficial- Spreading	41	0.96	0.77	0.96	0.70	0.82	0.91	0.77	0.87	0.90
*P*-value (one-sided)			0.09	0.31	0.15	0.28	0.17	0.59	0.32	0.81	0.09
BRAF mutation	Y	25	0.93	0.75	0.93	0.65	0.82	0.85	0.77	0.87	0.89
	N	33	0.90	0.70	0.93	0.72	0.70	0.82	0.68	0.9	0.82
*P*-value (one-sided)			0.13	0.45	0.36	0.44	0.06	0.16	**0.04**	0.49	0.13
NRAS mutation	Y	6	0.94	0.78	1.01	0.66	0.67	0.91	0.72	1.02	0.85
	N	45	0.94	0.75	0.95	0.75	0.79	0.85	0.71	0.87	0.88
*P*-value (one-sided)			0.28	0.40	0.41	0.31	0.38	0.37	0.40	**0.05**	0.39
BRAF or NRAS mutation	Y	31	0.93	0.75	0.94	0.65	0.82	0.89	0.77	0.90	0.89
	N	19	0.90	0.73	0.93	0.73	0.73	0.82	0.68	0.84	0.83
*P*-value (one-sided)			0.37	0.46	0.37	0.49	0.16	0.12	**0.02**	0.29	0.14

The statistical test used was the Wilcoxon two-sample test, unless otherwise stated (*Spearman correlation, **Kruskal-Wallis test).

Loss anywhere in the *CDKN2A* region was associated with ulceration (*P* = 0.002). However, ulceration of the tumor was most significantly associated with reduced gene dosage at *CDKN2B* (one sided test *P* = 0.006, two sided *P* = 0.01) and regions coding for P14ARF (one sided *P* = 0.002, two sided *P* = 0.004). There was some evidence of an association between ulceration and reduced gene dosage at *CDKN2A* coding for CDKN2A, *MTAP*, and the interferon gene cluster (Table 1).

**Figure 3:**
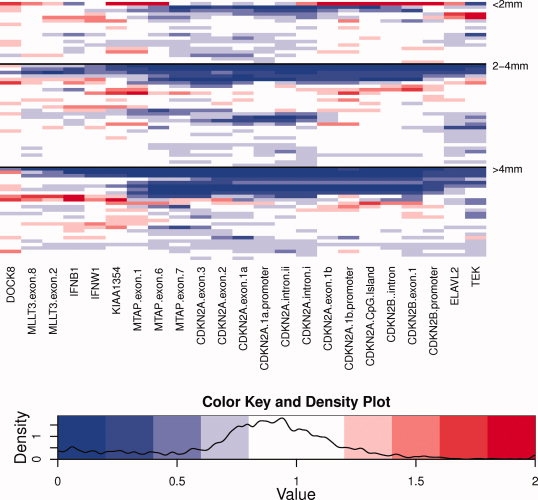
Heatmap of gene dosage at chromosome region 9p21 by Breslow thickness. Gene dosage ratios are shown for 18 tumors with Breslow thickness <2 mm (above), 30 tumors with Breslow thickness 2–4 mm (middle) and 27 tumors of thickness >4 mm. Blue coloration indicates reduced gene dosage, red coloration indicates increased gene dosage.

Relative loss across the region was associated with increasing tumor thickness (Fig. 3). Spearman correlation coefficients are given for thickness and reduced gene dosage across the *CDKN2A* region (Table 1). The most significant correlation was with reduced gene dosage at *CDKN2B* (*P* = 0.007 one-sided test) but there was some association across 9p21.3 (*P* = 0.02). To summarize, at 9p increasing thickness correlated with loss impacting on the coding regions of CDKN2B, CDKN2A, but not with loss in intronic regions of *CDKN2A*, *MTAP* or the interferon gene cluster. Increased mitotic rate was associated with reduced gene dosage across *CDKN2A* and especially in regions coding for P14ARF and CDKN2A (one sided *P* = 0.0006 and 0.004, respectively). There was no association between reduced gene dosage at 9p and tumor site or melanoma subtype (Table 1).

There was no significant association between *BRAF* and *NRAS* mutation status and reduced gene dosage in the *CDKN2A*/*CDKN2B* regions with the Wilcoxon Two-Sample test, but there was a trend toward an association between the presence of *BRAF* mutations and loss at regions coding for CDKN2A (one sided *P* = 0.06, two sided *P* = 0.12). The absence of *BRAF* mutation was associated with reduced gene dosage at *MTAP* (one sided *P* = 0.04, two sided *P* = 0.09) and the absence of an *NRAS* mutation was (borderline) associated with reduced gene dosage at the interferon gene cluster (one sided *P* = 0.05, two sided *P* = 0.10).

### *BRAF* and *NRAS* Mutations

Seventy-one tumors were successfully assayed for common mutations activating the MAPK pathway in melanoma, i.e., *BRAF* exon 15 and *NRAS* exon 2 mutations. Seventy-seven percent of relapsers had *NRAS*/*BRAF* mutation compared with 58% of nonrelapsers (Fisher's exact *P* = 0.17). There was no association between *NRAS*/*BRAF* mutation and mitotic rate or presence of ulceration (Fisher's exact *P* = 0.42), nor with thickness (*P* = 0.97). 71% of non-acral tumors had a mutation compared with 50% of acral tumors (*P* = 0.25). There were a slightly higher proportion of superficial spreading tumors than nodular tumors with a mutation (78 and 60%, respectively).

### Expression of CDKN2A in Primary Melanomas

**Figure 4:**
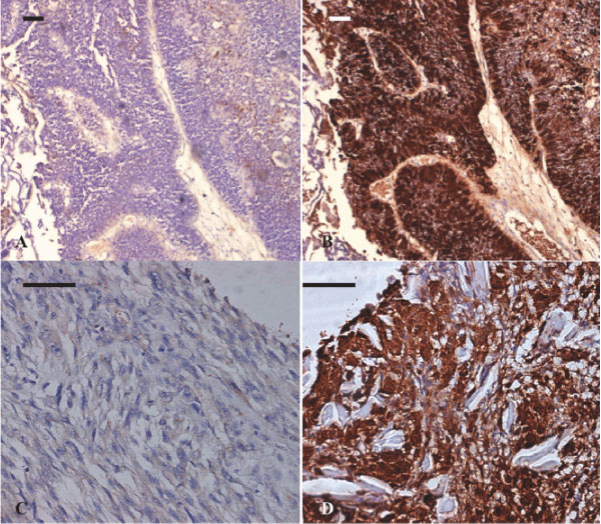
Immunohistochemistry of CDKN2A in primary tumors. (A) Bladder tumor control minus primary antibody. Some nonspecific stromal staining is visible in the stroma. (B) Strong specific cytoplasmic and nuclear staining in a bladder positive control in presence of primary antibody. (C) Results from representative tumors show very low cytoplasmic staining of CDKN2A from a tumor with a large homozygous deletion of *CDKN2A*. (D) Strong nuclear and cytoplasmic staining of CDKN2A and unstained normal stromal cells from a tumor which retained 2 copies of *CDKN2A*. Scale bar = 25 μm.

High specificity of staining was achieved in the control sections, where in the absence of primary antibody only haematoxylin staining is observed (Fig. 4A). In the presence of primary antibody, intense nuclear and cytoplasmic staining was observed for CDKN2A in some samples (Fig. 4B). In the 17 melanomas tested, the staining pattern varied from absent to strong cytoplasmic and nuclear staining. The level of positive expression of CDKN2A was associated with MLPA results of gene dosage in *CDKN2A* coding regions for CDKN2A in the majority of tumors (13/17) (Table 2). In the four samples with 80% gene dosage loss, there was a complete absence of CDKN2A expression in the region sampled for DNA extraction (Fig. 4C). In the seven samples with 50% gene dosage loss of the intronic, promoter or coding regions of *CDKN2A*, there was moderate or absent CDKN2A expression in four samples, while the remaining three showed strong CDKN2A expression. In the six samples that showed normal gene dosage at *CDKN2A* 5/6 showed strong to moderate CDKN2A expression (Fig. 4D) and one showed no expression of CDKN2A.

**TABLE 2:** Summary of CDKN2A Protein Expression Results Determined by Immunohistochemistry

Sample	Relapse	*CDKN2A* gene dosage	CDKN2A expression
1	N	≥ 80% loss intron/promoter	0
2	Y	≥ 80% loss whole gene	0
3	Y	≥ 80% loss whole gene	0
4	Y	≥ 80% loss whole gene	0
5	Y	50% loss promoter	2
6	N	50% loss intron	0
7	N	50% loss intron/gene	1
8	N	50% loss gene	2
9	N	50% loss intron	2
10	Y	50% loss intron	0
11	Y	50% loss intron/promoter	0
12	Y	Normal	2
13	N	Normal	2
14	N	Normal	0
15	N	Normal	2
16	Y	Normal	2
17	Y	Normal	1

Intensity of staining was scored as absent (0), expressed (1), or highly expressed (2). *CDKN2A* gene dosage determined by MLPA analysis is shown for each tumor sample.

## DISCUSSION

We have examined the role of *BRAF* and *NRAS* mutations and reduced gene dosage at 9p in melanoma relapse. The study was carried out using formalin-fixed paraffin-embedded (FFPE) tumors because of the difficulties in accessing cryopreserved primary tumors; melanomas are generally too small to allow cryopreservation. In the study, 65% of tumors sampled yielded enough DNA for MLPA screening and 61% were successfully sequenced for *BRAF/NRAS* mutations.

The observation that the MLPA and sequencing failure rate was higher in samples with lower DNA quantity indicates a potential for bias toward successful sampling of thicker, poorer prognosis tumors. However, failure rate was comparable in both relapse and nonrelapse groups, and over 40% low DNA input samples gave reliable results.

We found deletion (reduced gene dosage) at the *CDKN2A* locus to be common in primary melanoma. This is consistent with previous studies which have shown that deletion at *CDKN2A* appears to be the major mechanism of CDKN2A inactivation in primary melanomas (Funk et al.,^1998^; Fujimoto et al.,^1999^; Rizos et al.,^1999^; Cachia et al.,^2000^; Straume et al.,^2002^; Zhang and Rosdahl,^2004^). We did not screen for mutations as previous studies have consistently shown small sequence alterations to be uncommon (Ruiz et al.,^1998^; Cachia et al.,^2000^). Epigenetic silencing of *CDKN2A* by methylation (Gonzalgo et al.,^1997^; von Eggeling et al.,^1999^; Straume et al.,^2002^) could not be investigated in this study due to constraints on DNA availability from small tumors. However, protein expression of CDKN2A by immunohistochemistry correlated well with gene dosage ratios as determined by MLPA in the majority of tumors investigated, and is consistent with the view that deletion remains the dominant method of *CDKN2A* silencing in primary melanoma.

Reduced gene dosage impacting on CDKN2A was predictive of relapse. Loss impacting on P14ARF was, however, more strongly associated with poor prognostic factors such as increased Breslow thickness but particularly with mitotic rate and ulceration, than was loss impacting on CDKN2A. This suggests that loss of P14ARF has a key role in the progression of melanoma. This is consistent with our recent work in metastases, which demonstrated the loss of P14ARF by methylation or deletion to be common in metastases (Freedberg et al.,^2008^). This is the first study to report on loss across the critical region of 9p in primary melanoma, although loss of *CDKN2A* coding for CDKN2A has been suggested by others to be associated with poorer prognosis (Cachia et al.,^2000^; Grafstrom et al.,^2005^) and loss of CDKN2A expression immunohistochemically correlates with histological invasion (Talve et al.,^1997^; Pavey et al.,^2002^). Furthermore, the study suggests that within vertical growth phase melanoma, although loss impacting on CDKN2A was most frequent, wider deletions involving P14ARF and even CDKN2B were associated with poorer histological prognostic factors. Loss of CDKN2A is a common occurrence even in early melanoma (Tran et al.,^2002^). It is perhaps not surprising then, that further loss of the second melanoma tumor suppressor gene at 9p (P14ARF) impacts on outcome. There is in vitro evidence for a tumor suppressive role for CDKN2B in melanoma (Ha et al.,^2008^; Peters,^2008^; Schlegel et al.,^2009^). That reduced gene dosage impacting on CDKN2B also correlates with poor histological characteristics is supportive of the view that it too may play a role in tumor suppression in melanocytes.

One previous study has suggested that *BRAF* and *NRAS* mutations are less common in primary tumors with allelic loss on 9p (Kumar et al.,^2003^), and other studies using microsatellite markers located in the *CDKN2A* locus showed both *BRAF/NRAS* mutation and LOH at *CDKN2A* (Rodolfo et al.,^2004^). We showed no significant association between the presence of either *BRAF* or *NRAS* mutations and reduced gene dosage at 9p. Mutations in the *BRAF/NRAS* genes were not significantly associated with ulceration or Breslow thickness in these tumors. These observations are consistent with the view that these mutations are an early event in melanoma, required for initiation but not involved in progression (Platz et al.,^2008^), although there is one study which suggested that *BRAF* and *NRAS* mutations are more frequent in cells of the vertical growth phase of a melanoma than in the radial growth phase suggesting a selection for cells containing the mutations (Greene et al.,^2009^). Certainly, *BRAF* or *NRAS* mutations present in the primary tumor were found in the majority of associated metastases in another study (Edlundh-Rose et al.,^2006^). It is likely that secondary events are necessary for metastatic progression in addition to mutations in either *BRAF* or *NRAS*, but this could occur through alteration of other tumor suppressor genes or oncogenes not investigated here, such as *CDK4*, *PTEN*, and *TP53*, as well as *CDKN2A*. As only melanomas with wild-type *BRAF* have amplified CDK4 and cyclin D1 genes, the CDKN2A-CDK4/6-cyclin D pathway is viewed as linearly downstream of *BRAF* (Zhao et al.,^2008^). Inhibition of mutated *BRAF* using new specific *BRAF* inhibitors is reported in 2009 (ASCO) to be effective so that whether *BRAF* is a prognostic marker or not, *BRAF* driven MAPK pathway activation clearly drives tumor growth in melanoma tumors.

*ANRIL*, an antisense noncoding RNA, has recently been identified at 9p21.3 (Pasmant et al.,^2007^). The first exon of *ANRIL* is located in the *CDKN2A* P14ARF promoter, and the gene overlaps *CDKN2B*. There is evidence that *ANRIL* may influence transcription of the *CDKN2A* and *CDKN2B* genes (Jarinova et al.,^2009^; Liu et al.,^2009^). The frequency of deletion of *ANRIL* in primary melanoma tumors could not be investigated in this study as the MLPA assay used does not include probes specific for the *ANRIL* gene.

Deletion of *MTAP* is of interest as evidence for reduced expression in poor prognostic tumors has been reported before (Behrmann et al.,^2003^) and there is some evidence that loss may moderate response to interferon therapy (Wild et al.,^2007^). In this study, however, we showed no evidence for a role for *MTAP* in melanoma prognosis. We did see an association between reduced gene dosage at *MTAP* and the absence of a *BRAF* mutation, although the significance of this observation remains to be established.

In conclusion, we have identified a high frequency of deletion in the *CDKN2A* gene in primary melanoma tumors, which supports previous evidence that gene deletion is the major mode of inactivation of CDKN2A and bypass of cell cycle control required for proliferation and progression to metastatic disease in malignant melanoma. Reduced gene dosage at 9p (but not *BRAF* or *NRAS* mutation) was associated with histological features predictive of a poorer prognosis. The study also suggests that loss of P14ARF has an additional role in melanoma relapse and possibly also a role for CDKN2B coded by *CDKN2B*.
